# Patient perceptions of the use of e-cigarettes in smoking treatment programs: a qualitative analysis

**DOI:** 10.1186/s13722-025-00575-w

**Published:** 2025-05-30

**Authors:** Sidney V Rojas, Kelly A. Kyanko, Rachel Wisniewski, Katherine O’Connor, Rina Li, Grace Xiang, Mahathi Vojjala, Olivia Wilker, Scott E. Sherman, Elizabeth R. Stevens

**Affiliations:** 1 Department of Population Health, NYU Grossman School of Medicine, New York, NY USA; 2 New York University School of Global Public Health, New York, US

**Keywords:** Smoking cessation, E-cigarettes, NRT, Smoking behavior, Dual use, Nicotine

## Abstract

**Background:**

E-cigarettes may serve as a safer alternative to combustible cigarettes and may be more effective than currently available nicotine replacement therapy (NRT). Little is known about the perceptions of using e-cigarettes as part of a smoking treatment program. The objective of this study was to gain insight into patient-level factors to consider when developing smoking treatment programs that incorporate e-cigarettes.

**Methods:**

Qualitative analysis of in-depth interviews with 14 participants enrolled in the e-cigarette treatment arm of a tobacco treatment intervention pilot randomized trial comparing the impact of behavioral counseling paired with e-cigarettes or NRT on smoking outcomes. Participants were prompted to share their experiences with the products and the study overall. Transcripts were coded according to the principles of framework analysis for applied research. Codes were organized into themes using the principles of grounded theory.

**Results:**

Themes suggest that while there is an eagerness to try e-cigarettes as a new tool for smoking cessation, there is apprehension regarding what it means to “quit” if switching to e-cigarettes. Reflecting on the transitional purpose of e-cigarettes and potential health concerns associated with their use, many participants differentiated between the short-term goal to quit combustible cigarettes and the long-term goal to quit e-cigarettes.

**Conclusions:**

Including e-cigarettes as an option in smoking treatment regimens may be an opportunity to re-engage people who smoke who have tried and failed to quit with other forms of treatment. Participants found it challenging to establish what it means to quit cigarettes with e-cigarettes due to addiction and other health concerns. Clear guidelines are needed for integrating e-cigarettes into smoking cessation programs.

**Trial Registrations:**

ClinicalTrials.gov Identifier: NCT04465318.

**Supplementary Information:**

The online version contains supplementary material available at 10.1186/s13722-025-00575-w.

## Background

Smoking continues to be the leading cause of preventable death and disease in the United States (US) [1] and there is an ongoing need for effective smoking cessation tools. While not approved as a quit smoking aid by the US Food and Drug Administration (FDA), electronic cigarettes (e-cigarettes) are increasingly being used for smoking cessation [2]. Evidence suggests that the use of e-cigarettes containing nicotine is associated with increased quit rates [3–5]. E-cigarettes may serve as a useful addition to currently available FDA approved nicotine replacement therapy (NRT). However, there is limited guidance available as to best practices for integrating the use of e-cigarettes into smoking treatment [6]. 

To guide the incorporation of e-cigarettes into smoking treatment programs, there is a need to understand patient-level factors that may impact program adoption and effectiveness. Qualitative studies tend to focus on the general acceptability of e-cigarettes among users and non-users [7–11]. These studies offer insights about consumer preferences and habits, but are not as helpful for understanding the motivations and behaviors of those who switch to e-cigarettes to quit smoking. More research is needed to understand perceptions of those who use e-cigarettes for the primary purpose of quitting smoking [12]. 

The decision-making process to switch from combustible cigarettes to e-cigarettes has been particularly complex to characterize [13]. In addition to varying intent of e-cigarette use, there is a diverse range of product types and use patterns [14, 15]. Dual use of e-cigarettes and combustible cigarettes is increasingly common among those trying to quit smoking [16]. However, research shows that there is much uncertainty among dual users, primarily in regard to the health risks and benefits of dual use [17]. To better understand these diverse use patterns and address patients’ concerns, further qualitative analysis is needed.

In an effort to better understand the motivations and ambiguity of e-cigarette usage for smoking cessation, this analysis explores users’ attitudes and behaviors regarding the transition between combustible cigarettes and e-cigarettes. By examining the perspectives of research participants enrolled in a tobacco treatment intervention pilot randomized controlled trial (RCT), this analysis offers insight into patient-level factors to consider when developing smoking treatment programs that include e-cigarettes.

## Methods

To assess the acceptability of a smoking intervention and determine points for potential program improvement, a qualitative analysis was performed of in-depth interviews conducted with participants of an RCT pilot study comparing the effect of behavioral counseling with the use of e-cigarettes versus behavioral counseling with NRT on change in smoking outcomes including smoking cessation and switching to e-cigarettes. The RCT had an intervention period of 12 weeks in which participants received up to 6 smoking counseling sessions and an as-needed supply of NRT or e-cigarettes. The primary outcome, change in cigarette use, was collected via self-report [18, 19]. The study protocol was approved by the New York University Langone Health (NYULH) IRB (#s20-00839) and written documentation of informed consent was obtained prior to data collection.

### Setting and participants

Participants were recruited via a convenience sample upon completion of the intervention phase of the RCT at the 12-week follow-up study visit. All RCT participants were invited to participate in an in-depth interview to discuss their experiences with the intervention and other aspects of the study. Recruitment was ended once thematic saturation was reached. Participants were provided with a $20 incentive for their participation in the interview portion of the study.

The pilot RCT was performed to determine the feasibility and acceptability of an e-cigarette-based smoking intervention, and to compare the effectiveness of counseling + e-cigarettes or counseling + NRT on smoking outcomes [19]. RCT participants were recruited from the electronic health record (EHR) of NYULH, a private non-profit hospital system serving New York, New Jersey, and Connecticut. The RCT participant sample was initially restricted to patients with a diagnosis of chronic obstructive pulmonary disease (COPD), but the scope was later expanded to include patients with a diagnosis of coronary artery disease (CAD), peripheral artery disease (PAD), or asthma. In addition, to be eligible RCT participants were required to: smoke four or more days a week with at least five cigarettes on the days they do smoke; be motivated to quit smoking; and possess a phone with text messaging capabilities. A total of 121 participants were recruited into the pilot RCT. Participants in the RCT e-cigarette arm who completed an interview were included in this qualitative analysis.

### Data collection

Semi-structured in-depth telephone interviews lasting approximately 30 min were conducted between April 2021 and November 2022. The interviews were intended to assist with further adaptation of the e-cigarette smoking intervention and behavioral counseling manual used in the RCT. All interviews were conducted by an experienced team member trained in interviewing (OW). The interview guide was developed based on the pilot RCT procedures to provide additional insights about participant experiences, intentions to quit, barriers and facilitators to e-cigarette use, and how to refine the current approach to enhance program retention and outcomes. Interviews covered topics such as program aspects the participants liked/disliked; features of the intervention that should be modified; their experiences using e-cigarettes/NRT; intentions of using e-cigarettes after the intervention; and whether their heath symptoms interfered with their ability to engage in the intervention.

In the interview, participants were prompted to discuss multiple aspects of e-cigarette use. Along with follow-up probes, the participants were asked: “Have you noticed any positive effects from e-cigarette use?”; “How about any negative effects from e-cigarette use?”; “Do you believe the use of e-cigarettes has helped you reduce your cigarette intake?”; “How likely will you continue using e-cigarettes in the future? And why?”; “What are your thoughts about e-cigarettes in comparison to combustible cigarettes?”; “Do you think e-cigarettes are a good way to quit or cut back on smoking? And why do you think so?”; and “Overall, what do you like or not like about e-cigarettes?” A complete interview script is available under Additional file 1.

### Data analysis

Interviews were audio-recorded, transcribed, and imported into Dedoose software for qualitative data analyses. Interview transcripts were coded using procedures designed to ensure thoroughness and reliability. We coded data according to the principles of the ‘framework for applied research,’ [20] which consists of a five-stage process including familiarization, identifying themes, indexing, charting, and interpretation. Codes were primarily developed a priori based on intervention components and the quality improvement goals of the study, which were oriented by a user-centered design framework [21]. Additional codes were developed by reviewing a random sample of interviews and discussion with the coding team. The general development of themes arose from the data, using the principles of grounded theory [22]. To enhance reliability, two researchers took part in the coding and analysis process for each interview. Prior to full coding, a random sample of interviews were double coded and assessed for intercoder reliability via percent agreement (range: 79.5–87.9%). All disagreements were discussed as a group to improve concordance among coders. All interviews were then independently coded by a combination of two of five coders (GX, KO, RL, RW, SR) who met with the other coders and co-author (ERS) to resolve discrepancies. Coders were research team members who were trained in qualitative coding and specifically the application of the developed code book. When coding was complete, the outputs of quotations with each code were examined, summarized, and grouped together into themes.

## Results

### Participants

Thirty out of 63 (48%) RCT participants in the e-cigarette arm were invited to participate in interviews. Four declined to participate, one interview was not included in the analysis due to audio malfunction, and 11 interviews were ultimately not scheduled due to the research team’s perceived achievement of thematic saturation. Fourteen interviews were included in this analysis. The participants included in the analysis had an average age of 58 (range: 28–74), 36% identified as female, 64% were White, 21% were Black, 7% were Hispanic, and 7% were Asian. The majority (71%) of participants had completed at least some college, 29% had a high school education or less. Two-thirds of participants (64%) had a diagnosis of COPD, 21% of participants were diagnosed with CAD/PAD, and 14% of participants were diagnosed with asthma. Compared to e-cigarette arm RCT participants who did not complete the interview, those who completed the interview were more likely to be unemployed (2% versus 20%), but did not differ significantly in age, gender, race, or educational attainment.

### Themes

Through the analysis of the interviews, three major themes emerged to characterize the experiences of participants using e-cigarettes for smoking cessation: (1) Optimism for a new smoking cessation tool, (2) e-cigarettes as an intermediary for switching away from combustible cigarettes, and (3) grappling with what it means to quit. Within those themes, subthemes were identified regarding the attributes of e-cigarettes and how they compare to combustible cigarettes, dual use and substitution, definitions of quitting using e-cigarettes, as well as concerns about health effects and replacing one habit with another.

#### Optimism for a new smoking cessation tool

Participants expressed an eagerness to try a new tool for smoking cessation as an alternative to previously unsuccessful or uninteresting quitting methods. When speaking about their efforts to reduce their cigarette consumption throughout the study period, one participant attributed much of their success to the optimism provided by this new tool, saying, “I believe that there is a level of hope that, [the use of e-cigarettes] created” (Participant E04). Participants spoke about their lack of success with other smoking cessation products and their eagerness to try something new. One participant said that the opportunity to try e-cigarettes is “…why I joined the study… having tried the patch and the gum, unsuccessful cold turkey…” (Participant E34). Another participant continued on to emphasize their interest in trying the e-cigarettes as a novel smoking cessation method, stating “I would always say, ‘I wanna do the patches’ or ‘I wanna do the gum.’ But, I never attempted to try. But, this one I wanted to try” (Participant E14).

#### E-cigarettes as an intermediary for switching away from combustible cigarettes

##### Attributes of e-cigarettes

Participants frequently spoke about the qualities of e-cigarettes that made them an effective intermediary for switching away from combustible cigarettes. As one participant stated, “I got my nicotine fix through the electronic cigarettes” (Participant E09). For another participant, they saw e-cigarettes as a “perfectly acceptable substitution” to combustible cigarettes (Participant E05). In addition to addressing the body’s physical addiction to nicotine, many participants discussed how e-cigarettes acted as a helpful intermediary to address the habitual mental aspects of smoking:

“It’s just an alternative thing that you’re smoking. And I feel that’s an easier way to get people to step away from cigarettes. A lot of the time when people want to quit, they just go do something completely different. And that’s kind of forcing the body or the brain to do things quicker than it may want to or you may be able to. So it doesn’t take out the option of smoking if you’re trying to quit. It helps with weaning.” (Participant E48).

In particular, and distinct from NRT, the e-cigarettes helped with the “weaning” process by giving participants an alternative to hold, pull on, and inhale. As several participants explained, paired with the “inhale feeling” of the e-cigarette, “it was much easier than the patches and the gum because you just had something to do with your hands” (Participant E20).

##### Dual use and substitution

Also reflective of the transitional characterization of e-cigarettes, many participants discussed their patterns of e-cigarette and combustible cigarette dual usage and substitution. While the exact pattern varied between individuals, participants commonly cited that they started smoking fewer combustible cigarettes and more e-cigarettes as the program went on. As one participant described, “if you could replace a combustible cigarette with an electronic cigarette, it certainly cut down on because there was one less combustible cigarette that I would have” (Participant E09). Some participants tried to completely switch, but more often would go “back and forth” between combustible cigarettes and e-cigarettes (Participant E04). This was also regarded as a redeeming characteristic because it demonstrated “I can actually talk myself out of saying I want the cigarette” (Participant E48). For others, it reflected the fact that e-cigarettes would never compare to combustible cigarettes. As one participant explained, “I go back to the e-cigarettes just out of guilt and then I go back to combustible cigarettes out of necessity to fulfill some stress level or because I couldn’t get the same draw from the e-cigarette and it didn’t comfort me the way that a combustible cigarette would” (Participant E04).

#### Grappling with what it means to quit

##### Definitions of quitting using e-cigarettes

Within the “back and forth,” transition between combustible cigarettes and e-cigarettes, participants’ goals for replacing and/or substituting combustible cigarettes varied, along with their definitions of “quitting.” Some participants defined quitting as “ceasing all inhalation products, whether it be tobacco or vapor based” (Participant E24). Other participants only included combustible cigarette smoking cessation in their goals and definition of quitting, while remaining opening to e-cigarette usage. One participant explained, “I set myself up a quit date where I said…I’m going to start just using the vape pen and not buying cigarettes anymore” (Participant E20). Combining these approaches, several people differentiated between their short-term goal to quit combustible cigarette smoking and long-term goal to quit e-cigarette smoking as well. These individuals tended to characterize e-cigarettes as more of a holdover: “I saw [e-cigarettes] as a bridge to help eliminate the combustible and eventually for they themselves to be eliminated” (Participant E09). Also referred to as a “tool” or a “crutch,” one participant was unsure about e-cigarette usage going forward: “I’m not sure about getting off the e-cigarette, and maybe now that might be my goal in the future too, but I feel like I found that this is like a step in helping me to quit cigarettes” (Participant E14).

##### Concerns about health effects and replacing one habit with another

Within the various smoking cessation frameworks, an important consideration among participants was worry about the health effects of e-cigarettes and replacing one addiction with another. One participant summarized this, saying, “I’m going to be dependent on the e-cigarette getting me through to the other side. And once I get to the other side, meaning not smoking cigarettes, I’m going to be using these e-pens for quite a while” (Participant E34). Participants suggested that using e-cigarettes to quit combustible cigarettes was going to lead to e-cigarette dependency, an area with ambiguous health impacts. For another participant, “when [the e-cigarette] got to be almost as good as my cigarette, I was smoking just as many of them as I was of the cigarettes. And I don’t think I got to where I wanted to be. So, I needed to reevaluate” (Participant E04). However, while some people were “buying more e-cigarettes than [they] would like to” by the end of the program (Participant E01), others found the e-cigarettes to be less addictive:

“E-cigarettes helped me to reduce smoking the combustible and I did not get addicted to e-cigarettes… That’s what I was most worried about, is if I was going to get addicted to e-cigarettes. And I didn’t. To me, that’s the most positive thing.” (Participant E24).

## Discussion

Through the exploration of perceptions of the smoking treatment program among participants using e-cigarettes to quit smoking, various themes emerged. Themes suggest an optimism for a new smoking cessation tool and the perceived potential for e-cigarettes to serve as an intermediary for switching away from combustible cigarettes. However, the study also revealed the difficulties of grappling with what it means to quit when e-cigarettes are being used to reduce combustible cigarette use. This study suggests that the inclusion of e-cigarettes in a smoking cessation regimen might be perceived as beneficial, especially for combustible cigarette users who have found FDA approved forms of NRT to be less effective. However, the results of this study emphasize that when integrating e-cigarettes into smoking treatment, considerations will likely be needed to address patients’ concerns over e-cigarette addictiveness and safety, as well as to moderate expectations of e-cigarette functionality such as sensation, dosage, and other differences from using combustible cigarettes.

Including e-cigarettes as an option in smoking cessation NRT regimens may be an opportunity to re-engage people who smoke who have previously tried and failed to quit with FDA approved forms of NRT. Similar to observations made in previous qualitative research studies [23, 24], participants in our study tended to report their experiences with e-cigarettes as a comparison to currently available NRTs. In particular, participants suggested that e-cigarettes provide a source of optimism for achieving smoking cessation after experiencing previous ineffectiveness and side effects associated with FDA approved types of NRT. This suggests that including e-cigarettes as an additional option within the NRT toolbox may encourage individuals to make additional quitting attempts. Additionally, the qualities of e-cigarettes themselves may contribute to their appeal as a smoking cessation device [24]. Participants highlighted the unique features of e-cigarettes that enabled them to address both the nicotine addiction and behavioral habits of smoking, describing how e-cigarettes simulate combustible cigarettes to serve as both a nicotine fix and as a replacement product to inhale and puff on.

Our findings raise concerns over the potential for dual use of e-cigarettes and combustible cigarettes during quit attempts. While still being studied, health risks of dual use do exist [25, 26]. As dual use is often prominent among users attempting to transition from combustible cigarettes to e-cigarettes [11], smoking treatment programs including e-cigarettes are likely to need an additional educational counseling component to discourage individuals from using both products simultaneously. However, there are indications that dual use may be nuanced with multiple types of tobacco use transition patterns leading to varying levels of reduced smoking [27], as well as varying levels of health risks [25, 26]. For instance, compared to gradually transitioning from combustible cigarettes to e-cigarettes, abruptly switching has been associated with longer durations of abstinence from smoking [28]. Further research is needed to understand if any other patterns of dual use positively influence quit rates and abstinence duration. Importantly, these findings could establish if complete transition from combustible cigarettes is the only acceptable quit pattern to encourage.

Similarities between e-cigarettes and combustible cigarettes and the addictive nature of e-cigarettes can make it difficult for users to establish what it means to quit smoking when also using e-cigarettes. This difficulty was observed as participants indicated their differing understandings of what “quitting” truly means to them. While several participants saw e-cigarettes as a potentially helpful “bridge,” “tool,” or “crutch,” to stop combustible cigarette use, they lacked consensus about if and how to wean off e-cigarettes in the future. Even within an individual, e-cigarettes and “quitting” can be perceived differently depending on the perceived short-term and long-term roles of e-cigarettes.

For some participants, complete nicotine abstinence was the only true “quit.” Many saw e-cigarettes as simply an addiction replacement for combustible cigarettes or they perceived continued use of e-cigarettes as a potential gateway to slide back into combustible cigarette use. This concern over e-cigarettes as a “slippery slope” that possibly threatens smoking cessation, has been observed previously among people who smoke and those who used to smoke alike [29]. However, other participants expressed less concern over how and if they would be able to stop using e-cigarettes in the future. As previously observed in ex-smokers who currently use e-cigarettes [30], this suggests a lower sense of urgency to quit nicotine once they switch to e-cigarettes as compared to when using combustible cigarettes. Although e-cigarettes may serve as an important harm-reduction strategy, continued e-cigarette use is not completely without risk [31]. Thus, for some, smoking treatment programs are likely to need additional components to encourage similar abstinence from e-cigarettes.

As medical practitioners consider recommending e-cigarettes as a smoking cessation aid [32], more clarity is needed to define the role e-cigarettes may play in a smoking treatment regimen. While many regard NRT as a medical product, research shows that the uses and implications of e-cigarettes are perceived to be more ambiguous [29]. In the absence of clear guidelines for using e-cigarettes for smoking cessation, aspects of their function and use remain ambiguous and confusing to users—how to track usage [33] compared to combustible cigarettes, risk factors [34] and long term health effects of using e-cigarettes, and best practices for transitioning from combustible cigarettes to e-cigarettes. The Centers for Disease Control (CDC) recognizes e-cigarettes’ potential to aid adults who smoke to quit smoking and discourages dual use if used as a quitting aid, but their recommendations lack concrete detail [6]. To address these sources of ambiguity, further efforts are needed to solidify the role of e-cigarettes in smoking treatment guidelines.

This study had a few limitations. First, while thematic saturation was achieved, the final sample size of e-cigarette users interviewed was relatively small and only included individuals willing to try using e-cigarettes, therefore limiting the conclusions that can be drawn from these analyses. Future research should seek to understand the perspectives of a larger and more diverse sample of people who smoke, including those who have stated they are not open to using e-cigarettes. Second, the sample of interview participants was a convenience sample and the interview was not required, but rather was offered to all participants sequentially as an optional component of the RCT. This potentially introduced selection bias, as those with stronger opinions of the program may have been more likely to participate. Similarly, one inclusion criteria for participation in the RCT was a willingness to use e-cigarettes. This likely biased the sample population towards those with an existing interest in e-cigarettes.

## Conclusion

Including e-cigarettes as an option in smoking cessation NRT regimens may be an opportunity to re-engage people who smoke who have previously tried and failed to quit with FDA approved forms of NRT. However, due to their addictive nature and potential health effects with the use of e-cigarettes it can be difficult to establish what it truly means to quit smoking. To reduce confusion among consumers and establish best practices, further efforts are needed to develop guidelines for the effective incorporation of e-cigarettes into NRT programs.

## Electronic supplementary material

Below is the link to the electronic supplementary material.

Supplementary Material 1

## Data Availability

The datasets analyzed during the current study are available from the corresponding author upon reasonable request.

## Abbreviations

NRT: Nicotine replacement therapy
FDA: US Food and Drug Administration
RCT: Randomized controlled trial
NYULH: New York University Langone Health
IRB: Institutional Review Board
EHR: Electronic health record
COPD: Chronic obstructive pulmonary disease
CAD: Coronary artery disease
PAD: Peripheral artery disease
CDC: Centers for Disease Control
